# Evaluation of Anxiety Levels Following Treatment-Related Information in Patients Undergoing Orthognathic Surgery

**DOI:** 10.3390/healthcare14213696

**Published:** 2026-11-01

**Authors:** Ömer Faruk Kocamaz, Meyra Durmaz, Mehmet Alp Eriş, Mahzun Yıldız, Berivan Deniz, Merve Berika Kadıoğlu, Mehmet Emre Yurttutan

**Affiliations:** 1 Department of Oral and Maxillofacial Surgery, Faculty of Dentistry, Ankara University, 06560 Ankara, Türkiye; omerf.kocamaz@gmail.com (Ö.F.K.); mehmet.alp.eris@gmail.com (M.A.E.); mzunyildiz22@gmail.com (M.Y.); bdeniz@ankara.edu.tr (B.D.); memreyurttutan@yahoo.com (M.E.Y.); 2 Department of Orthodontics, Faculty of Dentistry, Ankara University, 06560 Ankara, Türkiye; mkadioglu@ankara.edu.tr

**Keywords:** orthognathic surgery, patient counseling, preoperative anxiety, State–Trait Anxiety Inventory

## Abstract

**Background:** Preoperative patient education is an essential component of orthognathic surgery. This study aimed to compare short-term changes in anxiety following separate orthodontic and surgical information consultations in patients scheduled for orthognathic surgery. **Methods:** This prospective observational study included 27 patients scheduled for bimaxillary orthognathic surgery. Participants attended standardized information consultations with an orthodontist and, approximately two weeks later, an oral and maxillofacial surgeon. Anxiety was assessed using the State–Trait Anxiety Inventory (STAI) and a visual analog scale (VAS). Trait anxiety (STAI-T) was assessed at baseline, state anxiety (STAI-S) was measured after each consultation, and VAS scores were recorded immediately before and after both consultations. The primary outcome was the within-participant difference between surgical and orthodontic VAS changes, analyzed using the Wilcoxon signed-rank test. Secondary comparisons were adjusted using the Holm method. **Results:** The mean age was 23.44 ± 5.20 years, and 59.3% of participants were female. VAS scores did not change significantly following orthodontic consultation (3.59 vs. 3.56; *p* = 0.891), whereas they increased significantly following surgical consultation (3.96 vs. 5.93; *p* < 0.001). The mean VAS changes were −0.04 ± 1.06 and 1.96 ± 1.29, respectively. The direct comparison of these changes demonstrated a significant difference between consultations (mean paired difference: 2.00 ± 1.59; *p* < 0.001). The mean baseline STAI-T score was 45.07 ± 5.89. Post-consultation STAI-S scores were higher after surgical than orthodontic consultation (46.96 ± 4.86 vs. 43.85 ± 6.24; *p* = 0.008; Holm-adjusted *p* = 0.015). **Conclusions:** VAS anxiety scores remained unchanged following orthodontic consultation but increased after surgical consultation, with a significant difference between the within-session changes. However, the fixed consultation sequence and proximity of the surgical consultation to the operation prevent causal attribution of these differences to consultation type. These findings support consideration of patients’ psychological responses throughout multidisciplinary preoperative care.

## 1. Introduction

Orthognathic surgery comprises surgical procedures performed in conjunction with orthodontic treatment to correct severe dentofacial deformities in individuals who have completed their growth and developmental phases. This approach is utilized to enhance masticatory function, alleviate temporomandibular joint disorders, and improve facial aesthetics [1,2]. The treatment process comprises preoperative orthodontic preparation, surgical intervention, and postoperative orthodontic retention phases, and requires close collaboration between the orthodontist and the oral and maxillofacial surgeon (OMFS) [2].

Although serious postoperative complications are rare, issues such as bleeding, sensory loss or paralysis due to nerve injury, infection, temporomandibular joint disorders, and relapse may occur following orthognathic surgery [3,4]. Due to these potential complications, patients experience anxiety not only about the aesthetic and functional outcomes but also regarding the recovery process and potential risks. Issues such as dietary restrictions, temporary interruption of school or sports activities, pain management, and oral hygiene constitute major sources of concern for patients and their families. To improve the attitudes of patients and their relatives, the surgical team should provide education about postoperative discomfort, surgical outcomes, risks, and the overall care process. Sharing this information preoperatively contributes to increased patient satisfaction [5].

Patient satisfaction is one of the primary objectives of this treatment. It is not sufficient for patients to be satisfied solely with the final surgical outcome. Particularly in a procedure of such elective nature, patients must be well informed and adequately prepared [6]. Indeed, it has been emphasized that dissatisfaction often stems not from the quality of the surgical outcome, but from inadequate patient information and counseling [7]. The most frequently reported causes of dissatisfaction include insufficient information regarding jaw fixation, lack of explanation about areas affected by postoperative nerve injury, and inadequate clarification of issues related to diet, swelling, pain, and scarring [8].

The concept of preoperative anxiety was first defined by Ramsay. It is described as a state of worry and tension experienced by the patient due to factors such as illness, hospitalization, anesthesia, surgical intervention, or uncertainty [9]. Such anxiety is observed in 25% to 80% of patients prior to surgery and may adversely affect the recovery process [10]. Anxiety may weaken the immune system, delay wound healing, and consequently prolong recovery time by increasing the release of stress hormones such as cortisol and adrenaline. Psychological stress has also been associated with behavioral and somatic manifestations, including bruxism and temporomandibular symptoms [11,12]. Indeed, studies have demonstrated that high levels of preoperative anxiety are associated with longer hospital stays [13,14]. Moreover, it has been reported that high levels of preoperative anxiety may increase the risk of severe postoperative pain, complications, and even morbidity and mortality in certain types of surgery [10,15]. Therefore, preoperative anxiety is considered an important and preventable risk factor for postoperative complications.

Patient education prior to orthognathic surgery is one of the fundamental responsibilities of both the OMFS and the orthodontist [16]. Differences in clinical knowledge and patient counseling attitudes between these two specialties have also been reported in the context of temporomandibular disorder management [17]. As the orthodontist spends more time with the patient throughout the treatment process, they have the opportunity to establish a trust-based relationship. Therefore, the orthodontist’s responsibility to explain the treatment planning, process, scope of surgery, and possible outcomes is of great importance [18]. The OMFS should provide a detailed explanation of the scope of the procedure, its risks, potential complications, and the recovery process [19]. Although there are studies in the literature evaluating the effect of preoperative information on patient anxiety, no research has been identified that assesses the difference in anxiety levels resulting from separate information provided by the orthodontist and the OMFS [20,21,22].

The aim of this study was to compare anxiety responses following separate preoperative information sessions provided by the orthodontist and the OMFS in patients scheduled for orthognathic surgery and to contribute to the improvement of patient education processes.

## 2. Materials and Methods

### 2.1. Study Design and Ethical Approval

This was a prospective observational study with a within-subject repeated-measures design. It was conducted in collaboration between the Departments of Orthodontics and Oral and Maxillofacial Surgery at the Faculty of Dentistry, Ankara University. Data collection took place between April and September 2025. Ethical approval was obtained from the Ethics Committee of Ankara University (Approval Date: 14 April 2025; Approval No: 8/27). The study was carried out in accordance with the ethical principles and guidelines of the Declaration of Helsinki. All participants were provided with verbal and written information about the study, and informed consent forms were obtained. The study was prepared in compliance with the STROBE (Strengthening the Reporting of Observational Studies in Epidemiology) guidelines for reporting observational research.

### 2.2. Participants

The study included individuals scheduled for bimaxillary orthognathic surgery who had completed preoperative orthodontic treatment, had no history of trauma to the jaw or facial region, no neurological or systemic diseases, no syndromic or congenital craniofacial deformities such as cleft lip and palate, were not on regular neurological or psychiatric medication, had not undergone previous orthognathic surgery, and had no mental disorders that could interfere with participation in the study.

Individuals with a history of trauma to the jaw or facial region; complaints of pain, discomfort, or functional limitation in any part of the body; cleft lip and palate, syndromic craniofacial anomalies, or congenital deformities; a history of orthognathic or major facial surgery; or diagnosed systemic, neurological, or psychiatric disorders were excluded from the study.

**Figure 1:**
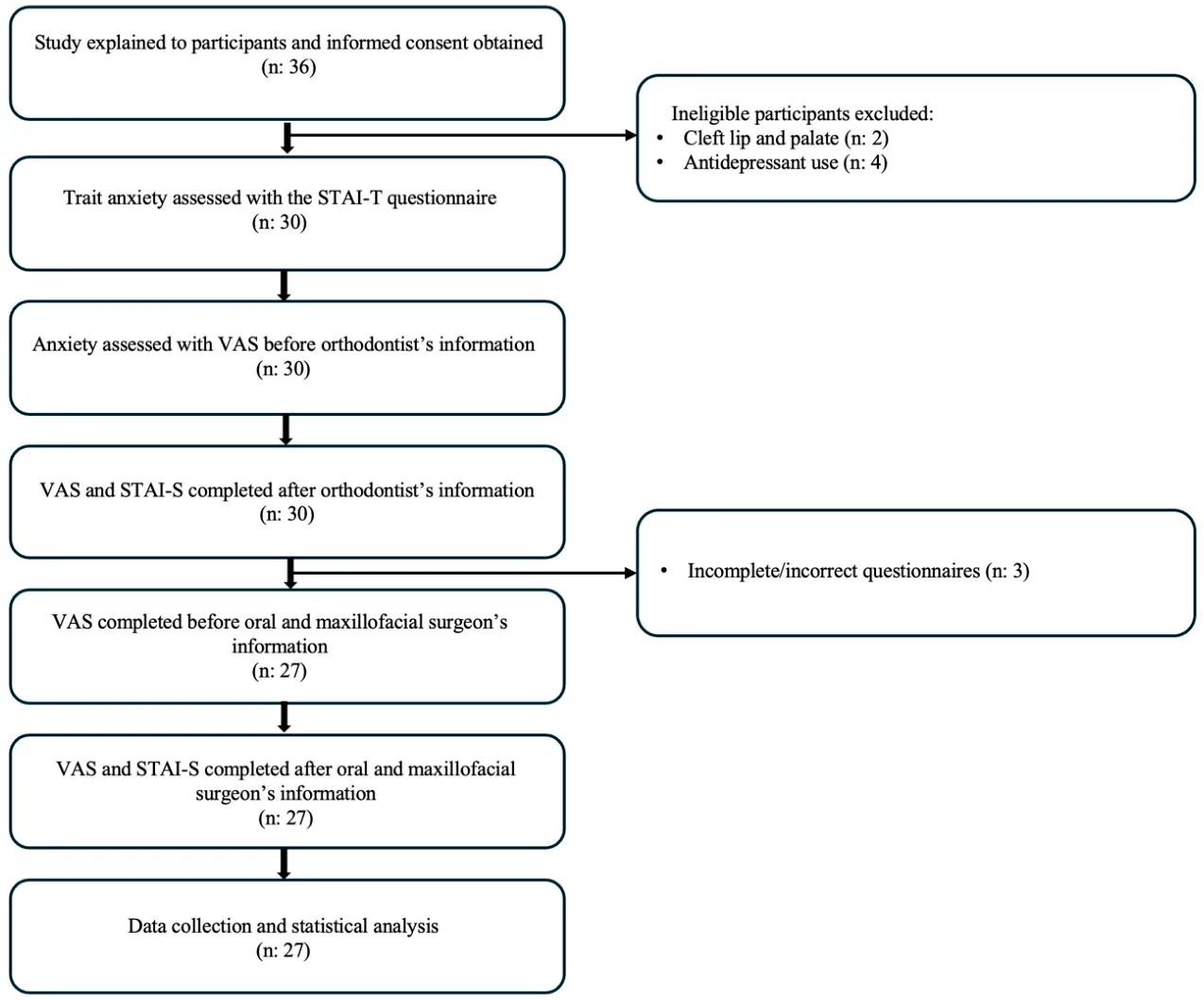
Flowchart of the study.

A total of 36 individuals were invited to participate in the study. Two were excluded due to the presence of cleft lip and palate. Four participants were excluded from the analysis because of regular antidepressant use. Additionally, three participants, despite completing the forms during the orthodontic information phase, refused to complete the questionnaires following the surgical information session. Consequently, data from 27 participants were found to be complete and eligible for analysis, resulting in a valid response rate of 75.0% for the study (Figure 1).

The sample size was calculated using G*Power 3.1 software based on a one-tailed paired-samples t-test. In the absence of directly comparable preliminary effect-size estimates, a medium effect size (Cohen’s dz = 0.50) was assumed according to Cohen’s conventional benchmarks [23]. With α = 0.05 and 80% power, the required minimum sample size was 27 participants. A supplementary two-tailed sensitivity analysis indicated that the final sample of 27 participants could detect an effect size of dz = 0.56 with 80% power. As the calculations were based on paired comparisons rather than repeated-measures ANOVA, ANOVA-specific parameters were not incorporated.

### 2.3. Questionnaire and Measurement Instruments

The data collection form used in this study consisted of three main sections.

The first section contains demographic and health-related information such as participants’ age, sex, regular medication use, and use of antidepressants.

The second section of the questionnaire, the State–Trait Anxiety Inventory (STAI), developed by Spielberger, was used to assess participants’ anxiety levels [24]. The inventory consists of 40 items divided into two subscales. Items 1–20 form the State Anxiety Inventory (STAI Form TX-S/SAI), which evaluates individuals’ levels of anxiety, tension, restlessness, or apprehension regarding current or recent situations or potential future events. Items 21–40 comprise the Trait Anxiety Inventory (STAI Form TX-T/TAI), which measures general and persistent anxiety tendencies, reflecting personality-based anxiety levels. The scale employs a 4-point Likert-type rating system, ranging from 1 (almost never) to 4 (almost always). Higher total scores indicate higher levels of anxiety. The Turkish validity and reliability study of the STAI was conducted by Öner and Le Compte (1983) [25]. The Turkish version of the scale underwent linguistic and cultural adaptation, its psychometric properties were confirmed, and it has been recognized as a reliable instrument for assessing anxiety levels in the Turkish population.

In the third section of the questionnaire, a 10-point Visual Analog Scale (VAS) was used to assess participants’ subjective anxiety levels. The VAS is a unidimensional self-assessment tool that allows individuals to rate the intensity of subjective experiences such as anxiety, pain, or discomfort on a scale from 0 to 10. A score of “0” represents no anxiety, while “10” indicates an unbearable level of extreme anxiety. Participants were asked to mark the value that they believed best reflected their current level of anxiety.

### 2.4. Information Process and Application Timelines

Information sessions were conducted, and questionnaires were administered to a total of 27 orthognathic surgery patients who had completed their preoperative orthodontic treatment with the same orthodontist and were approximately four weeks away from surgery. Anxiety assessments were conducted at five time points. Data obtained from the participants were collected sequentially to evaluate the effect of the information processes on anxiety levels.

All questionnaires were administered in a quiet environment free from distractions, under the supervision of the same researcher, and accompanied by a standardized written information form. This form included details about the preoperative preparation process, the purpose and scope of orthognathic surgery, the techniques to be used, potential risks and complications (such as bleeding, infection, nerve injury, edema, and relapse), the postoperative recovery period, dietary and hygiene recommendations, and the post-treatment follow-up plan. In both clinics, the information sessions were conducted with identical content, tone, and duration to ensure standardization across measurements.

At the beginning of the study (T_0_—baseline), approximately one week before the orthodontic information session, participants completed the STAI-T (Trait Anxiety Form). This form was used to assess individuals’ general tendency toward anxiety.

Immediately before each information session conducted by the orthodontist and the OMFS, the VAS was administered to assess the participants’ subjective anxiety levels at that moment (T1 and T3).

Subsequently (T2), participants received information from the orthodontist, after which the STAI-S (State Anxiety Form) and the second VAS forms were administered. Approximately two weeks later (T_3_), the same participants completed only the first VAS form prior to receiving information from the OMFS. Finally (T_4_), following the OMFS’s information session, the STAI-S and second VAS forms were administered again (Table 1).

The VAS assessments performed immediately before and after each information session enabled the evaluation of short-term changes in anxiety within the same consultation. This approach allowed changes associated with each session to be assessed relative to the patient’s anxiety level immediately before receiving information, rather than relying solely on comparisons between the two consultation dates.

Through this five-stage design, data related to both the orthodontic and surgical information sessions were collected sequentially from each participant. The primary outcome was defined as the difference between the within-session changes in VAS scores following the orthodontic and surgical information sessions, calculated as (T4 − T3) − (T2 − T1). Post-consultation STAI-S scores at T2 and T4 were compared as a secondary outcome, whereas baseline STAI-T scores at T0 were used solely to describe participants’ general anxiety tendencies. Because STAI-S was administered only after each information session, within-session changes in STAI-S scores could not be evaluated.

### 2.5. Statistical Analysis

Data analysis was performed using SPSS Statistics 26.0 (IBM Corp., Armonk, NY, USA). Descriptive statistics were presented as mean ± standard deviation, median (minimum–maximum), and percentage (%). Data normality was assessed using the Shapiro–Wilk test. The Mann–Whitney U test was used for comparisons between gender groups. The primary analysis compared within-session changes in VAS scores (post-session minus pre-session) between the orthodontic and surgical information sessions using the Wilcoxon signed-rank test. Secondary analyses included within-session VAS comparisons before and after each information session and the comparison of STAI-S scores obtained after the two information sessions, using the Wilcoxon signed-rank test. The Holm method was applied to adjust for multiple testing across these three secondary comparisons. Baseline STAI-T scores were evaluated descriptively and were not compared with STAI-S scores. All statistical tests were two-sided, and *p* < 0.05 was considered statistically significant.

## 3. Results

A total of 27 individuals were included in the study. Of the participants, 59.3% were female (*n* = 16) and 40.7% were male (*n* = 11), with a mean age of 23.44 ± 5.20 years (Table 2).

No statistically significant difference was found between sexes in terms of STAI and VAS scores (*p* > 0.05). Female participants showed a higher tendency for pre-orthodontic information VAS scores compared to males (4.38 ± 2.80 vs. 2.45 ± 2.34), although this difference was not statistically significant (*p* = 0.073). The effect size of this tendency was moderate (r = 0.32), suggesting that women may experience relatively higher subjective anxiety during the pre-surgical period. No sex differences were observed at other time points (T0, T1, T2, T3, T4) (*p* > 0.05) (Table 3).

The mean baseline STAI-T score was 45.07 ± 5.89, reflecting participants’ general anxiety tendencies. The mean STAI-S scores following the orthodontic and surgical information sessions were 43.85 ± 6.24 and 46.96 ± 4.86, respectively. A statistically significant difference was observed between the post-consultation STAI-S scores, with higher state anxiety levels recorded following the surgical information session (*p* = 0.008; Holm-adjusted *p* = 0.015) (Table 4).

Examination of the VAS scores revealed no significant difference between pre- and post-orthodontic information sessions (3.59 vs. 3.56; *p* = 0.891; Holm-adjusted *p* = 0.891). In contrast, a statistically significant increase was observed following the surgical information session (3.96 vs. 5.93; *p* < 0.001; Holm-adjusted *p* < 0.001). The mean VAS changes were −0.04 ± 1.06 and 1.96 ± 1.29 following the orthodontic and surgical information sessions, respectively. Direct comparison of these within-session changes revealed a statistically significant difference between the two information sessions (*p* < 0.001) (Table 5).

## 4. Discussion

This study investigated short-term changes in anxiety levels before and after orthodontic and surgical consultations in a homogeneous cohort of bimaxillary orthognathic surgery candidates. Sequential measurements using VAS and STAI documented preoperative anxiety levels revealed changes in anxiety scores across the consultation periods.

High preoperative anxiety in surgical populations, including orthognathic surgery patients, may negatively affect postoperative outcomes. Meta-analyses and reviews have reported associations with increased pain and analgesic requirements, higher incidence of postoperative nausea and vomiting, and delayed recovery [26,27]. In addition, a large cohort study demonstrated that patient-reported anxiety is associated with increased postoperative morbidity [28]. In summary, the findings indicate that preoperative anxiety—particularly in third molar surgery—may increase postoperative complaints; however, consistent results have not been observed across all studies regarding pain outcomes [29,30]. Therefore, incorporating preoperative anxiety screening into the care pathway and providing targeted informational or psychoeducational interventions is clinically rational. Indeed, structured informational consultations in oral and maxillofacial surgery patients have been shown to reduce both preoperative anxiety and early postoperative discomfort (e.g., pain, nausea, vomiting) [22]. Moreover, postoperative nausea and vomiting are common issues in oral and maxillofacial surgery, underscoring the importance of thorough risk assessment and management [31]. In our study, anxiety fluctuations accompanying the examination and information stages were analyzed separately, and it is recommended that anxiety be systematically considered in preoperative care planning.

Preoperative anxiety in orthognathic surgery candidates is closely associated with the patient’s level of knowledge, uncertainty, and expectation management [20]. Randomized findings indicate that multimedia-based or structured informational interventions can reduce anxiety levels [22,32]. Furthermore, since the quality of information content and the framing of risks can influence patients’ perceptions and satisfaction, enhancing the quality of communication is important for clinical outcomes [19,33]. In our study, anxiety levels remained relatively stable following orthodontic information, whereas an increase was observed after surgical information. Although both consultations followed a standardized protocol with identical information content, differences in the clinical setting and patients’ familiarity with the healthcare professionals may have contributed to the observed anxiety responses. However, these potential influences were not directly evaluated and should be interpreted cautiously.

Several studies in the literature have reported high levels of preoperative anxiety among orthognathic surgery candidates. For instance, Zhou et al. (2024) reported median preoperative STAI-S and STAI-T scores of 39 and 40, respectively, in a series of 371 cases, identifying clinically significant anxiety (STAI-S ≥ 40) in approximately half of the patients (47.7%) [34]. Similarly, Kok et al. reported preoperative anxiety levels in orthognathic surgery patients [20]. However, comparisons with previous studies should distinguish between trait and state anxiety, as STAI-T and STAI-S measure different constructs and their scores are not directly interchangeable.

In our study, the mean baseline STAI-T score was 45.07, while the mean post-consultation STAI-S scores were 43.85 following orthodontic information and 46.96 following surgical information. Because STAI-T and STAI-S assess different dimensions of anxiety, these values should not be interpreted as changes from baseline. Although both consultations followed a standardized protocol with identical information content, tone, and duration, higher state anxiety scores were observed after the surgical consultation. This difference may reflect the clinical context, patients’ perceptions of the upcoming operation, or the timing of the consultations rather than differences in the information provided. One possible interpretation draws on Lazarus and Folkman’s stress-coping model [35], which suggests that individuals’ appraisal of a situation may influence their emotional responses. The surgical consultation may have been perceived as more threatening because of its clinical context or proximity to the operation. However, patients’ cognitive appraisals were not directly assessed in this study; therefore, this explanation remains hypothetical.

Patients may perceive the OMFS as a central figure in surgical decision-making, whereas the orthodontist may represent continuity and familiarity throughout the longer course of treatment. These differences in perceived professional roles and patient–clinician relationships could potentially influence emotional responses during consultations. Patient perceptions of oral and maxillofacial surgeons have also been shown to vary according to aspects of professional appearance, including clinical attire [36]. Previous studies have suggested that empathetic communication may help reduce patient anxiety and improve perceived control over treatment [37,38]. However, perceived familiarity, trust, communication dynamics, and patient–clinician relationships were not independently assessed in the present study. Therefore, their potential contributions to the observed anxiety differences remain hypothetical.

Differences in clinical setting and patients’ perceptions of surgical consultations may also contribute to variations in anxiety responses. For example, an unfamiliar environment or the anticipation of surgery could potentially increase perceived uncertainty and emotional distress [39]. However, environmental familiarity, perceived safety, and physiological stress responses were not evaluated in this study. Therefore, these factors should be considered possible explanations rather than established mechanisms underlying the observed differences.

The orthodontic information session was conducted approximately two weeks before the surgical consultation, which took place closer to the operation date. As surgery approaches, patients may experience greater anticipatory anxiety, potentially influencing their responses during preoperative consultations [39,40]. Therefore, the higher post-consultation STAI-S scores observed after the surgical information session may reflect differences in consultation timing and clinical context. However, the fixed sequence of consultations prevents these potential influences from being distinguished from the effects of the information session itself.

Studies examining the effect of sex on anxiety have yielded inconsistent results; some have reported higher scores among females [41], whereas larger series have found that sex is not an independent determinant, with preoperative psychological status and postoperative complications (pain, PONV, sleep disturbance) serving as stronger predictors [34]. In our sample, no significant differences were observed between sexes in STAI or VAS scores.

In this study, all patients received information from the same OMFS and the same orthodontist using a standardized protocol in terms of content and duration. This approach helped reduce variability in information delivery. However, the observed differences in anxiety may have also been influenced by consultation order, timing, and unmeasured patient-related factors. Furthermore, the single-center, single-provider design may limit generalizability. Given the observational nature of the study, these findings should not be interpreted as evidence of a causal relationship. Future multicenter studies with alternative consultation sequences and information formats may help clarify these associations.

Although VAS measurements obtained immediately before and after each consultation allowed the assessment of short-term changes in anxiety, the fixed sequence of consultations and the proximity of the surgical consultation to the operation may have influenced anticipatory anxiety. Therefore, the observed increase in anxiety cannot be attributed exclusively to the surgical information session.

## 5. Conclusions

Preoperative anxiety responses in patients undergoing orthognathic surgery may vary across different clinical consultation settings, even when standardized information is provided. In the present study, VAS scores did not change significantly following orthodontic consultation, whereas a significant increase was observed immediately after surgical consultation. These differences may be associated with consultation timing and clinical context; however, the fixed consultation sequence and observational design prevent causal interpretation. These findings highlight the importance of considering patients’ psychological responses throughout the multidisciplinary preoperative information process. Incorporating empathetic communication, clear explanations, and appropriate psychological support may help address patients’ anxiety during preparation for orthognathic surgery.

## Figures and Tables

**Table 1:** Measurement timeline and applied scales used in the study.

Timeline	Applied Scales	Description
T0 (Baseline)	STAI-T	The STAI-T was administered approximately one week before the information session and measured the individual’s general (trait) anxiety level.
T1	VAS-1	VAS-1 was administered immediately before the orthodontist’s information session to assess the participant’s momentary anxiety level.
T2	STAI-S + VAS-2	The momentary anxiety level was assessed immediately after the information session conducted by the orthodontist.
T3	VAS-1	The momentary anxiety level was measured before the surgical information session.
T4	STAI-S + VAS-2	The momentary anxiety level was assessed immediately after the surgeon’s information session.

**Table 2:** Age and sex distribution of the participants.

Sex	*n*	%	Age (Mean ± SD)	Median (Min–Max)
Female	16	59.3	23.12 ± 5.01	22.5 (17–36)
Male	11	40.7	23.89 ± 5.62	23.0 (18–40)
Total	27	100.0	23.44 ± 5.20	23.0 (17–40)

**Table 3:** Comparison of participants’ STAI and VAS scores by sex.

								Mann–Whitney U Test	
		*n*	Mean	Median	Minimum	Maximum	SD	Mean Rank	*p*
T0	Female	16	45.56	44.50	34.00	56.00	6.17	14.56	0.656
	Male	11	44.36	45.00	37.00	58.00	5.66	13.18	
T1	Female	16	4.38	5.00	0.00	9.00	2.80	16.25	0.073
	Male	11	2.45	2.00	0.00	7.00	2.34	10.73	
T2-STAI-S	Female	16	43.81	44.00	35.00	58.00	6.84	13.63	0.767
	Male	11	43.91	45.00	33.00	51.00	5.56	14.55	
T2-VAS-2	Female	16	4.19	4.50	0.00	9.00	2.71	15.81	0.149
	Male	11	2.64	2.00	0.00	7.00	2.54	11.36	
T3	Female	16	4.00	5.00	0.00	9.00	2.34	14.09	0.941
	Male	11	3.91	4.00	1.00	7.00	1.87	13.86	
T4-STAI-S	Female	16	47.69	47.00	39.00	58.00	4.57	15.19	0.345
	Male	11	45.91	46.00	38.00	56.00	5.30	12.27	
T4-VAS-2	Female	16	6.25	7.00	1.00	9.00	2.32	15.19	0.341
	Male	11	5.45	5.00	1.00	9.00	2.25	12.27	

T0: Baseline, T1: Before the orthodontist’s information session, T2: After the orthodontist’s information session, T3: Before the surgeon’s information session, T4: After the surgeon’s information session, *p*: Mann–Whitney U Test.

**Table 4:** Descriptive Statistics of Baseline Trait Anxiety and Post-Consultation State Anxiety Scores.

	*n*	Mean	Median	Minimum	Maximum	SD	T2–T4 STAI-S (*p*)	T2–T4 STAI-S (*p*-Holm)
T0 -STAI-T	27	45.07	45.00	34.00	58.00	5.89	0.008	0.015
T2-STAI-S	27	43.85	44.00	33.00	58.00	6.24		
T4-STAI-S	27	46.96	47.00	38.00	58.00	4.86		

T0: Baseline trait anxiety; T2: Following orthodontic consultation; T4: Following surgical consultation. *p*: Wilcoxon signed-rank test. Holm-adjusted *p*-values are reported for secondary comparisons.

**Table 5:** Comparison of VAS Scores Before and After Orthodontic and Surgical Information Sessions.

	n	Mean	Median	Minimum	Maximum	SD	*p*	*p* (Holm)
T1-VAS-1	27	3.59	4.00	0.00	9.00	2.75		
T2-VAS-2	27	3.56	3.00	0.00	9.00	2.71		
ΔVAS – Orthodontic	27	−0.04	0.00	-4.00	2.00	1.06	0.891	0.891
T3-VAS-1	27	3.96	5.00	0.00	9.00	2.12		
T4-VAS-2	27	5.93	7.00	1.00	9.00	2.29		
ΔVAS – Surgical	27	1.96	2.00	0.00	4.00	1.29	<0.001	<0.001
ΔVAS Surgical - ΔVAS Orthodontic	27	2.00	2.00	0.00	6.00	1.59	<0.001	-

VAS: Visual Analog Scale; ΔVAS: Change in VAS score; SD: Standard deviation. *p*-values were calculated using the Wilcoxon signed-rank test. Holm-adjusted *p*-values are reported for secondary comparisons.

## Data Availability

The raw data supporting the conclusions of this article will be made available by the authors on request.

## Abbreviations

The following abbreviations are used in this manuscript:

OMFS	Oral and maxillofacial surgery
STROBE	Strengthening the reporting of observational studies in epidemiology
STAI	State–Trait Anxiety Inventory
SAI	State Anxiety Inventory
VAS	Visual Analog Scale
STAI-T	Trait Anxiety Form
STAI-S	State Anxiety Form
